# Acute Needs, ACUTE Response: Development and Delivery of a Mental Health Urgent Care in the Bronx

**DOI:** 10.1007/s10597-024-01448-3

**Published:** 2025-01-09

**Authors:** Kahlil C. DuPerry, Shaina Siber-Sanderowitz, Elisabeth Hill, Melissa Cintron-Arroyo, Allison Glasgow, Julia Vileisis

**Affiliations:** 1 Department of Psychiatry and Behavioral Sciences, Montefiore Medical Center, Bronx, NY USA; 2 Department of Psychiatry and Behavioral Sciences, Albert Einstein College of Medicine, Bronx, NY USA; 3 Affirm Mental Health, Gilbert, AZ USA

**Keywords:** Access to care, Crisis intervention, Program development, Teleservices

## Abstract

As mental health needs rise, creative and timely solutions are essential. Leveraging the expansion and flexibility of virtual services to create telehealth and hybrid offerings is crucial for addressing systemic barriers in mental health, enhancing accessibility, and providing flexible, comprehensive care options for diverse patient populations. This article discusses the development of a mental health urgent care program within a large medical system in a densely populated, under-resourced community. The program was designed to address common community mental health barriers across multiple care entry points, including ambulatory settings, emergency care, and consultation services. Using a multipronged approach, this program aims to improve patient access, care continuity, and outcomes. The authors encourage others to consider adopting a similar programmatic infrastructure to reduce mental health care barriers in their communities.

The proliferation of urgent care mental health services has coincided with the ongoing mental health crisis following the COVID-19 pandemic (Du et al., 2021). While mental health services have always been difficult to access for people in under-resourced areas and with marginalized backgrounds, one of the biggest barriers to many has been months-long waitlists and limited means of access (Breslau et al., 2022). An urgent care mental health service delivery model, especially one that integrates telehealth capabilities, may be able to substantially lower the barriers to treatment that exist in some of the most at-need communities (Breslau et al., 2022; Du et al., 2021).

The Bronx, New York, the birthplace of Hip-Hop, is known for its vibrant culture influenced by waves of immigration from all over the world and migration throughout the city. It is one of only two of New York State’s 62 counties where non-White residents are the largest racial group with 54.8% of the population Hispanic or Latino and 28.5% of the population Black or African American (U.S. Census Bureau, 2022). With the Bronx being one of the hardest hit areas in one of the hardest hit cities during the COVID-19 pandemic, ranking 10th in number of deaths per county (Johns Hopkins, 2023), never before has the need for rapid mental health evaluation, delivery of care, and connection to broader services been higher.

The following article describes the development, delivery, and planned expansion of the Ambulatory Consults, Urgent Treatment, and Evaluations (ACUTE) clinic within a large hospital system in the Bronx, beginning with a brief review of mental health needs within the Bronx and an exploration of how mental health urgent care services have met the needs of various other communities. By describing the service that we offer to our patients, we hope to elucidate another possible way for other systems to meet the needs of often under supported and marginalized communities.

## Background

### Mental Health Needs in the Bronx

Mental health was always a need in New York City, but the COVID-19 pandemic exacerbated an already dire issue, with 45% of adults 18–34 reporting poor mental health and 25% of those with poor mental health reporting difficulty obtaining mental health support (NYC Health and Hospitals, 2022). This problem is particularly acute in the Bronx which ranked 62nd out of 62 counties in New York State for health (NYC Health and Hospitals, 2022). The Bronx has the lowest utilization of mental health resources by those in distress among the five boroughs, with only 43% of residents in distress are utilizing resources, which was statistically significantly lower than Manhattan (69%) and Staten Island (71%), and trending lower than Brooklyn (61%) and Queens (54%; Breslau et al., 2022). Responding to the mental health needs of the Bronx will require more easily accessible services.

### Mental Health Needs at an Academic Medical Center in the Bronx

The Psychiatry department of the academic medical center in question is comprised of four main areas of psychiatric care: outpatient, emergency, inpatient consult/liaison services, and inpatient psychiatry units. The development team spoke with leadership across the different psychiatry services to identify potential areas for intervention that would increase access to care and decrease barriers to care. All areas identified were related to high volume and stretched staffing, which often led to long wait-times and limited resources being over- or under-utilized. In order to support patients who may fall through the cracks of the existing services, a clinic for urgent and timely services was devised.

### Urgent Care Mental Health Service Delivery

Urgent care mental health services are a growing area of service delivery, however, there are limited studies describing their development and implementation. A cursory search using PsychInfo, PubMed, and GoogleScholar return limited results with recently published literature reviews coming to similar conclusions (see Coates, 2018; Du et al., 2021; Xie et al., 2022). What follows is a brief review of relevant literature on the structure, process, and outcomes of urgent care mental health services that influenced the development of a model within a large medical system in a densely populated, under-resourced community.

While many different structures of urgent mental health services exist, a consistent factor was the implementation of interdisciplinary teams (Du et al., 2021). Teams often consisted of a combination of nurses, psychologists, social workers, mental health counselors, psychiatrists, and internists (Alvarado et al., 2020; Du et al., 2021; Kroll et al., 2019). Many services existed outside of a hospital system, in the community and others were physically located within hospital departments (e.g., ED, outpatient clinic) and therefore able to leverage resources and collaborate with those departments (Alvarado et al., 2020; Coates, 2108; Du et al., 2021). While other examples exist, developing within a large hospital system meant modeling off of prior services that were co-located with Emergency Departments and Outpatient Departments, while adjusting to the unique needs of our community and system.

One of the primary goals of urgent care mental health services is to see patients who do not meet Emergency Department levels of risk sooner than a traditional outpatient clinic is often able to accommodate a new visit. Some services were able to see patients same-day for walk-in visits (Alvarado et al., 2020; Kroll et al., 2019) while others ranged from two to twelve days later (Sunderji et al., 2015). Additionally, there were differing approaches to the type of service provided, with some focusing on triage and assessment, and others providing a limited number (e.g., 6–8) of follow-up appointments (Alvarado et al., 2020; Kroll et al., 2019; Sunderji et al., 2015). Identifying high and low-risk patients and triaging them into appropriate workflows was also a common function (Stricker et al., 2018; Sunderji et al., 2015). There are very limited experimental designs to assess outcomes from urgent care mental health services, however what does exist points towards lowered barriers to treatment for those most in need including those on Medicaid, without a college degree, and more likely to miss appointments for various socioeconomic reasons (Du et al., 2021; Kroll et al., 2019).

## Development and Implementation of the Ambulatory Consults, Urgent Treatment, and Evaluations (ACUTE) Clinic

The ACUTE clinic is a new and developing clinic within the Psychiatry department of a large hospital system within the Bronx, New York. The clinic offers both timely (e.g., geriatric and pre-surgical evaluations) as well as urgent (e.g., bridge-care and virtual urgent care) services. We define “urgent” services as services that address mental health needs requiring immediate attention to prevent deterioration of the patient’s condition, but which do not rise to the level of emergency or crisis-level care filling the gap between the emergency department and routine outpatient services. For systems seeking to develop crisis response services, the SAMHSA (2020) guidelines would be more relevant to their goals, while the ACUTE model attempts to address less serious, though still important concerns, with fewer resources and upfront investment. “Timely” services are those that do not demand immediate attention and are not likely to be related to psychiatric deterioration, but without which may delay other care the patient is seeking.

The concept for ACUTE was initially proposed in the Fall of 2020. The Department of Psychiatry had undergone rapid changes in the wake of COVID-19 pandemic, accelerating the integration of telehealth services within ambulatory settings, providing an opportunity to address long standing access issues. Previously proposed solutions to access issues had to account for office availability and the physical location of both the patient and provider, creating silos in the system and hard limits in terms of expansion or integration of services. With many of these barriers reduced or eliminated with telehealth, there was a desire to create a multi-faceted service that functioned largely with telehealth, synergistically solving access issues within the department. Some of the access issues identified in interview with key stakeholders included: (1) reducing wait times for specialty evaluations, (2) providing care to patients who are not able to be seen by hospital outpatient clinics in a timely manner, (3) preventing lapses in medication adherence, and (4) reducing utilization of the psychiatric emergency department for urgent, though not emergent concerns. From the initial conceptualization phase through to the present, the intention was to create a clinic outside of the “full service” clinics that might not be able to provide the same level of wrap-around services, but would be able to be adaptable and move quickly to provide care wherever gaps were identified.

Delivery of services through the ACUTE clinic began with expansion of the traditional outpatient referral in 2020. Initial logistics steps included creating a separate program location within the electronic medical record (EMR), identifying regulatory parameters, establishing documentation and practice standards, and adding providers’ access. The earliest implementation included specialty consultations for geriatric patients with suspected or diagnosed age-related neuro-cognitive decline and gender diverse patients who required readiness evaluations for treatment. These services were operated with two psychiatrists and a clinical social worker as administrative lead and provider, eventually adding geriatric psychiatry fellows as well.

In the Fall of 2021 bridge care launched as a temporary telehealth service that would be offered until the client’s initial intake evaluation in the clinic. The initial launch of bridge care focused on identifying clients that were prescribed psychotropic medications but had been disconnected from care and would run out of their prescription prior to scheduled outpatient evaluation. Original bridge care staffing utilized unused provider capacity within the primary outpatient clinics without inflating caseload size in an unsustainable manner. Specifically, provider templates were updated to appropriately reflect their available clinical time versus their protected administrative, teaching, and research time. Providers with less than 85% clinical scheduling utilization were assigned at least one bridge care patient, more for those with lower utilization such as new staff psychiatrists who may only have 40% scheduling utilization as their caseloads are being built. As utilization grew, bridge care services were transitioned to a dedicated ACUTE clinic psychiatrist.

Further expansion and delivery of services were often disrupted during the COVID-19 pandemic as the hospital shifted focus to critical care and recovery efforts as waves continued. Around mid-2022, as the height of the pandemic had passed, attention was able to be refocused on development. As of writing, ACUTE is staffed by five Psychiatrists, one Social Worker, one Psychologist, and one Psychology Fellow. By having a growing interprofessional treatment team, ACUTE is able to offer patients the treatment that they need at the time that they need it. ACUTE providers work collaboratively with each other and the administrative team, in order to respond to gaps in services currently offered by ACUTE and other clinics.

In order to meet the needs of the system and to begin to address identified gaps, collaboration with established services, both within the Department of Psychiatry and in the broader hospital setting, was vital to the development of this new clinic. Cross-department collaboration often began at the individual relationship level with providers in other departments. Leveraging these relationships to identify known needs and build trust allowed for rapid development of workflows and implementation. While the process was slightly different for different services offered, largely the process followed four steps: (1) identify potential service partners and possible solutions to potential gaps in care that might be filled by ACUTE clinic; (2) meet with stakeholders to pitch potential solutions and identify other possible collaboration targets; (3) develop specific workflow to address the identified targets; (4) implement, evaluate, and update workflow with input from stakeholders. In the following sections, we describe the current services offered by the ACUTE clinic (Table 1).

**Table 1:** Brief description of ACUTE services

Track	Description	Key services
Behavioral Health Virtual Urgent Care (BHVUC)^1^	Same-day appoints accessible through patient facing electronic medical record system	- Brief psychiatric evaluation - Medication consultation - Therapeutic interventions and/or psychoeducation - Referral to appropriate follow-up options
Bridge Care^2^	Psychotherapy and medication provided to higher risk patients while awaiting intake for “full-service” clinic to limit likelihood of deterioration	- Medication initiation and/or continuation - Skills-based therapy - Co-ordination and warm hand-off with clinic
Specialty Consults^2^	Evaluations for access to and optimization of care	- Pre-surgical psychological evaluations (bariatric, gender affirming, spinal cord stimulator) - Emotional Support Animal letters - Geriatric psychiatric evaluations and follow up
Medication Management^2^	Medication appointments for patients who have graduated from therapy and are stable on medications and lower risk patients who have therapists outside of the “full-service” clinics	- Medication evaluations and continuation - Alternative to full d/c from clinic or PCP managing psychiatric medications - Alternative to changing therapists when medications are also indicated
Short-term Therapy^2^	Psychotherapy provided in a time-limited fashion focusing on clear and identifiable goals in an agreed upon timeline	- Skills-based and supportive therapy
Emergency Room Diversion^3^ (Discontinued)	Rapid discharge from Emergency Room for patients who do not have emergency-level concerns	- Rapid evaluation and referrals outside of emergency room context

^1^ Services provided exclusively through telehealth

^2^Services provided in person and through telehealth

^3^Services provided exclusively in person

### Specialty Consults

Previously, clients requiring psychological clearance for surgery, or other such one-time evaluations, were being referred to the same outpatient clinics that were providing long-term mental health treatment. This would lead to frustration for both providers and patients when a patient waited months for a clearance letter that the provider could not provide during the initial evaluation due to other intake related demands. The ACUTE clinic established a separate service pathway for patients and referring providers for evaluations. This served the dual purpose of expediting consults for those who typically had a time sensitive need and improving access to the outpatient clinics by removing specialty evaluation patients from referral queues. Specifically, providers could refer directly to the ACUTE service through the EMR or email and support staff were trained to identify main clinic referrals from the queue that would be better addressed by the ACUTE clinic. As more providers became aware of the ACUTE clinic, fewer referrals were made to the primary clinics for such evaluations.

### Geriatric Evaluations

As the population of older adults in the US rises, there is increasing demand for access to mental health care in this population despite the shortage of trained geriatric mental health providers to meet this need. In addition to requiring specialized care for primary psychiatric conditions, older adults are at greater risk of psychiatric manifestations of major neurocognitive disorders as well as other medical conditions. The prevalence of dementia, which disproportionately affects Black and Hispanic individuals, is 16.6% in the Bronx, compared with 12.7% in NY and 10% in the US, while the prevalence of mild cognitive impairment in the US is 22% (Dhana et al., 2023; Lee et al., 2022; Manly et al., 2022).

Given the limitations of clinics regulated by the New York State Office of Mental Health, often referred to as “Article 31 clinics,” patients with a primary diagnosis of neurocognitive disorder are considered ineligible for admission to such a clinic. However, many of the patients that would seek treatment at an Article 31 clinic would not be able to afford private practice psychiatrists. The Geriatric Psychiatry Specialty Consultation Service under the ACUTE Clinic serves to address this gap in care by providing medication management, psychoeducation, behavioral intervention, short-term therapy, advocacy for long-term care needs, one-time medication evaluation and consultation to primary outpatient teams, and caregiver support. This service also provides a medication track for patients who are no longer benefiting from long-term therapy in the primary clinics either due to cognitive impairment or stability of psychiatric symptoms, resulting in increased access to long-term mental health services by freeing up space in the primary clinic.

Through collaboration with care teams and community-based organizations, the consultation service provides comprehensive care to older adults. Furthermore, the Geriatric Psychiatry Service works with trainees including Geriatric Psychiatry Fellows, Psychiatry Residents, Medical Students, and Nursing Students to increase the knowledge and skills of professionals in caring for older adults.

### Gender Affirming Treatment Evaluations

It is estimated that approximately 1.6 million people age 13 and older identify as transgender in the United States, with an estimated 81,800 in the state of New York (The Williams Institute). Many of these individuals will pursue medical transition, which may require up to two letters of support from licensed mental health providers (Coleman et al., 2022). Getting mental health letters and readiness letters for surgery was one of the biggest barriers for gender diverse patients seeking gender affirming care, particularly for genital surgery (Tabaac et al., 2020). Through the specialty consult pathway we are able to reduce these barriers by creating an expediated referral pathway for readiness evaluations.

### Bariatric Evaluations

Given significant risks and a requirement for lifetime behavioral changes, bariatric surgery requires a psychological assessment to determine one’s appropriateness for surgery (Hubbard & Hall, 1991; Snyder, 2009). The weight loss surgery team at our institution conducts hundreds of bariatric surgery procedures a year, but did not have in house referrals for conducting the psychological assessment. These clients frequently found themselves on long waitlists in the behavioral health clinics or left to try to navigate outside referrals for this required assessment. At times difficulty getting connected to a provider who was able to perform this assessment would result in surgical procedures being delayed or canceled. ACUTE has been able to provide timely evaluations, with recommendations and treatment options for patients who meet criteria for eating disorders, as well as for those who would benefit from support making changes around sub-clinical disordered eating.

### Emergency Room Diversion

Due to a lack of urgent mental health options, it was not uncommon for patients to present to the Emergency Department for mental health services due to non-inpatient levels of distress or need for an outpatient appointment. Per discussions with staff, extended wait-times for such patients in the Emergency Department often lead to patients leaving prior to being seen or becoming agitated. As the average patient evaluation in the Psychiatric Observation Suite (POS) can take hours, an appropriate alternative would increase POS provider capacity to see other patients. ACUTE Psychiatry worked with POS leadership to develop a pathway to manage these patients in the outpatient clinic rather than through the Emergency Department.

When notified of a patient triaged to the waiting area, a marker for the lowest-risk patients, an onsite ACUTE provider would first review the patient’s chart. If appropriate, the provider would conduct a brief screening and safety assessment in the ED and obtain consent to be discharged to a same-day outpatient appointment. If the patient was deemed appropriate and consented, the ACUTE provider documented the brief evaluation, discharged the patient from the ED, walked them from the emergency room to the outpatient psychiatry department, then conducted a full initial evaluation. Patients who qualified for the track were those presenting looking for behavioral health referrals, medication refills, and those presenting with moderate distress (e.g., anxious or depressive symptoms) who were not suicidal, psychotic, or also experiencing significant medical complaints.

The program launched in October of 2022 with 2 providers offering 1 morning of diversion services each (9am – 1pm). Given low volumes (only 3 referrals in first 2 weeks) and the fact that most qualifying patients arrived later in the day, after 2 weeks the hours were extended to 9am – 4pm. Between October 2022 and February 2023, 14 patients were referred to the diversion program. Of these 14 patients, one required return to the emergency room for high acuity. Two patients left the emergency room between referral and seeing the diversion program care team. During the 4-month pilot, 11 patients were successfully diverted through the pathway, this 2–3 patient per month average was much lower than the estimated 20 patients per month expected. There are several reasons the program was under-utilized. The majority of patients who would be appropriate for this track may have presented to the ED during off-hours. Unfortunately, data on the timing of patients who came to the ED who would be appropriate for diversion were not collected. Additionally, it could be hard to tell from a patient’s chief complaint if they would be appropriate for the program. Therefore, patients may have received a full work-up in the ED who could have been seen by the diversion team. Given the low utilization and the need to direct resources to other programs, the program was discontinued in March 2023, transitioning resources to a behavioral health virtual urgent care.

### Behavioral Health Virtual Urgent Care

In 2019, the healthcare system launched an online virtual urgent care program to address common illnesses and minor injuries that was staffed by the Department of Emergency Medicine. This program, available to any patient who had an online account with the healthcare system, was an immediate success. For several years there had been discussion around the possibility of offering a similar program for behavioral health needs. Given the limited utilization of the ED diversion program, the decision was made to put resources that had previously been supporting this initiative to a new behavioral health virtual urgent care (BHVUC) initiative. The program launched in March of 2023 with 4 operating hours per day, staffed by a psychiatrist, psychologist, and social worker on different days throughout the week, one provider per day. The virtual walk-in clinic was initially opened without any external marketing efforts, still resulting in several initial visits within days of going live. To promote the program internally, a banner was placed on the landing page of the online portal, internal email communications announced the initiative, and posters and fliers were distributed in clinics throughout the hospital.

Exclusion criteria for care included patients under 18 years old, patients physically located out of state, patients who were already connected to active psychiatric care within the healthcare system, and patients who were having a psychiatric or medical emergency that would be more appropriately addressed by in-person emergency services. Prior to entering the virtual waiting room patients confirmed they were in the same state as the center, and a flag indicated that if they were experiencing a medical or psychiatric emergency, they should call 911 or seek in-person emergency services.

During the initial pilot phase, patient volumes were between one to three patients per day. Over the course of the first 6 months, 63 patients were seen through the platform. Volume was variable throughout the month, with a slight up-tick seen at the beginning and each of each month. The most common primary diagnoses were Depression (40%) and Anxiety (30%) Most visits were low acuity (86%), as defined by the clinical judgement of the providing clinician, with some moderate acuity patients (10%), and a small number of high acuity patients (5%); 2 patients required transfer to the ED. The majority of patients who presented were started on medication (65%) and resulted in a referral to long-term care (67%). If it was clinically appropriate, patients could receive follow-up care through the Bridge Care Treatment Track (see below).

The average appointment length was 52 minuteswith an average wait-time was 20 min The wait time was driven primarily by patient check-in process and trouble-shooting technical issues. Additionally, despite notifications informing patients, many patients were surprised by clinicians attempting to contact them after checking-in. Rather, many logged off of their patient account, expecting to have setup a future appointment, rather than seeing someone same-day.

### Bridge Care/Medication Treatment/Short-Term Therapy

#### Bridge Care

The Bridge Care Track can be divided into two major sub-tracks: medication management and bridge therapy. The medication management track has allowed providers within the hospital system to make referrals for patients with psychotropic medication needs to be seen and monitored while awaiting intake in a longer-term mental health setting. Similarly, bridge therapy allows for patients who may need added support while on a waitlist to be able to be seen for supportive and skill-based therapies in order to maintain safety and build positive coping strategies before beginning a course of therapy. Exclusionary criteria originally included: (1) the patient being on controlled substances or intermuscular medications, (2) the patient being actively suicidal, violent, or psychotic (as these patients receive expediated appointments), and (3) the patient could not be coming out of an emergency department or inpatient psychiatric setting. During the pilot the parameters were changed to allow for clients discharged from the Emergency Department to use bridge services as they were frequently not meeting criteria for an expedited appointment but often returned to the ED prior to an intake appointment. Additionally, collaboration with the Psychiatry Consultation/Liaison team has allowed patients coming from medical admissions to be identified for urgent visits as well. Bridge providers schedule patients based on their clinical determination, but a typical treatment course is 1–12 sessions over the two to 12 weeks prior to intake.

### Medication Treatment Track

In such a large health system, there are many mental health providers embedded in various clinics outside of the Psychiatry Department. Psychologists, Social Workers, and other non-prescribing providers embedded outside of the Department of Psychiatry often needed to share care outside of the system for patients with medication needs. When this was not possible, they had to make referrals to the primary clinics which, due to OMH regulations, meant therapy and medication needed to be provided by the primary clinic, even when the patient would rather work with the embedded provider, leading to many patients dropping out of care instead. The Medication Treatment Track was developed in order to provide medication only appointments for patients who would like to continue to be seen by their therapist, who often have specific specialties aligning with the patient’s needs (e.g., oncology, immigration, etc.), without having to split care outside of the system. Thus, engagement is able to be maintained as well as increasing the ability of the therapist and the prescriber to coordinate through shared EMR and other forms of communication. This pathway was designed for low acuity patients that could be safely managed outside the clinic environment. It also allowed for an opportunity for clinic patients to graduate from treatment and step-down to a less intensive treatment setting after meeting therapy treatment goals or when they were disinterred in therapy services. For patients who are in maintenance treatment and still benefit from psychotropic medication intervention this allows continuity of care.

### Potential Expansion and Next Steps

ACUTE plans to continue to grow and nimbly meet the needs of the healthcare system as identified through cross-service collaboration and discussion with internal partners. High demand for peri-partum psychiatric services has led to the recent addition of the Perinatal/Reproductive Mental Health Track. An unmet need for rapid access to care by hospital associates has led to a track to get hospital employees into care in a fast, confidential and comfortable way. A desire from patients and other providers for more appointment availability, both through telehealth and in-person, has led to staffing growth and ongoing consideration of a physical, dedicated space for the clinic. To date, ACUTE expansion has been driven by the needs of the hospital system and the needs of the community as understood by the hospital system. As the ACUTE model expands, we will also look directly to community partners to identify gaps and systemic blind spots.

To expand the ACUTE clinic while meeting new and more challenging needs, we have implemented informal quality improvement (QI) into all administrative meetings and have started to partner with other clinics in formal QI meetings when larger issues arise. We are tracking utilization of the services through completed encounters as well as outcomes of patients who utilize services. Plans are being made to further explore patient satisfaction and community impact and engagement.

## Discussion

The current article describes the development, implementation, and future directions of one model for a hospital system-based clinic for urgent and timely care. After an extensive development phase, exploring the gaps in services offered by the hospital system, a model that focused on Ambulatory Consults, Urgent Treatment, and Evaluations (ACUTE) was the best option for meeting the needs of our hospital system and the community it serves. Due to the impacts of the COVID-19 pandemic, development and implementation has been largely through a step-wise manner, allowing for new tracks to deliver services while others are still in development. Additionally, this has allowed for trial periods to be run, being able to see how estimates line up with reality, and allowing underutilized services to be disbanded without significant loss of resources. Other systems may find waiting to launch all services at once to be more applicable to their needs, or may focus on different tracks to develop to meet the needs of their communities. Further, the current model addresses gaps as they exist in our system within New York State. Given mental health regulations vary state-to-state, local laws and regulations should be considered when seeking to develop a mental health urgent care.

Addressing the ongoing mental health crisis both nationally and locally will require creative and flexible service delivery that is easily accessible. In order to respond to the ever-expanding needs of the community, mental health services that prioritize timeliness, targeted treatment, and preventative care will be vital. This article presents one possible model for development of an urgent care mental health service inside of a large health system that services one of the most diverse populations within the country with hopes that it may inspire others to do similar work in their own systems and communities.
